# A Case Series on Acute Mesenteric Ischemia (AMI) Leading to Intestinal Gangrene Following Blunt Trauma to the Abdomen

**DOI:** 10.7759/cureus.49092

**Published:** 2023-11-20

**Authors:** Aditya Sharma, Rahul Khanna, Ram Niwas Meena, Shashi Prakash Mishra, Seema Khanna

**Affiliations:** 1 Department of General Surgery, Institute of Medical Sciences, Banaras Hindu University, Varanasi, IND

**Keywords:** acute mesenteric ischemia (ami), blunt trauma, trauma surgery, bowel ischemia, upfront laparotomy

## Abstract

The term "acute mesenteric ischemia" (AMI) refers to a set of conditions where the blood supply to various segments of the small intestine is cut off, causing ischemia and subsequent inflammatory changes that might result in bowel gangrene. Estimates place the incidence between 0.09% and 0.2% of all acute surgical hospitalizations. Early diagnosis is essential, despite the entity being a rare cause of abdominal discomfort, because if left untreated, mortality is 50%. Herein, we present a case series of three patients with bowel ischemia following blunt abdominal trauma.

## Introduction

With some collateral flow from the celiac arterial system, via the superior and inferior pancreaticoduodenal arteries, as well as from the inferior mesenteric artery, the superior mesenteric artery (SMA) serves as the small bowel's main blood supply [1]. Depending on the physiological feeding state, the splanchnic circulation receives 15-35% of the cardiac output; nevertheless, oxygen extraction is rather low [2].

Therefore, before the small intestine becomes ischemic, the blood flow must be decreased by more than 50% [3]. Through improved oxygen extraction and perfusion brought on by vasodilation, the intestines can self-regulate their oxygen availability [4]. Mesenteric ischemia is not observed until the patient's mean arterial pressure falls below 45 mmHg [5]. A 75% drop in mesenteric blood flow can be sustained by the small intestine for up to 12 hours [6].

## Case presentation

Case 1

A 42-year-old male presented with diffuse acute abdominal pain and non-passage of flatus and stools following a road traffic accident. Except for the pulse rate of 116 per minute, his vital parameters were normal. Bowel sounds were sluggish, and abdominal discomfort and rigidity were present. His plain X-ray abdomen (erect posture) was normal. Ultrasonography showed dilated bowel loops with echogenic intra-peritoneal fluid. The patient was taken for an upfront laparotomy because of the peritoneal collection. CT was not done as the peritoneal signs warranted an urgent laparotomy.

**Figure 1:**
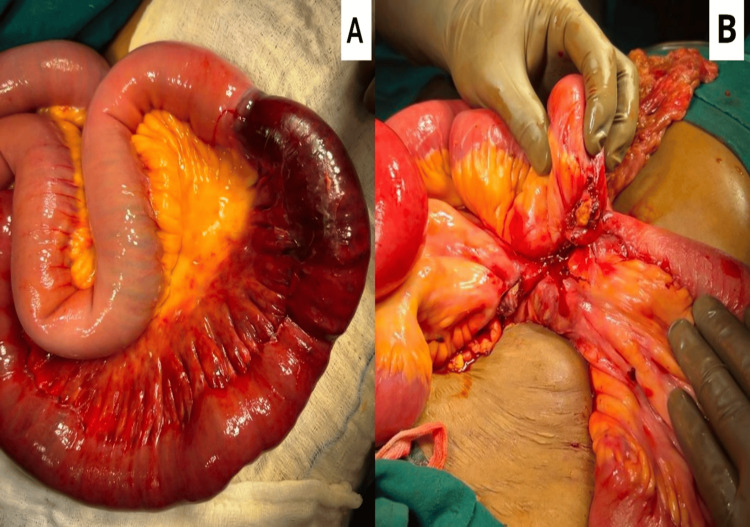
An intraoperative picture showing (A) a gangrenous ileal segment; (B) a resection anastomosis done.

Intraoperatively, approximately 3 feet of the ileal segment was gangrenous, and a bucket handle tear was noted in the small bowel mesentery, which led to the disruption of the arterial supply to the adjacent small bowel. Consequently, resection of the gangrenous small intestine with end-to-end anastomosis was done, as shown in Figure 1.

The postoperative period was uneventful. The patient was allowed oral intake on postoperative day six, which he tolerated well. He was discharged on day eight, and he did well during his follow-up visits to the hospital.

Case 2

A 32-year-old male presented with lower abdomen pain for the past two days. There were no disclosed co-morbidities or any previous surgery history. The patient's pulse rate was 122 beats per minute, and his blood pressure was 94/58 mmHg. Upon examination, the lower quadrant of the abdomen was tender, and bowel sounds were unremarkable. The complete blood count, including biochemical values, liver and kidney function tests, and PT/INR results, was all within normal limits. The X-rays of the chest and abdomen did not reveal anything unusual. Abdominal ultrasonography revealed the presence of echogenic collections in the peritoneal cavity. The patient underwent an exploratory laparotomy. A CT scan was not performed as the significant peritoneal signs warranted an emergent laparotomy.

**Figure 2:**
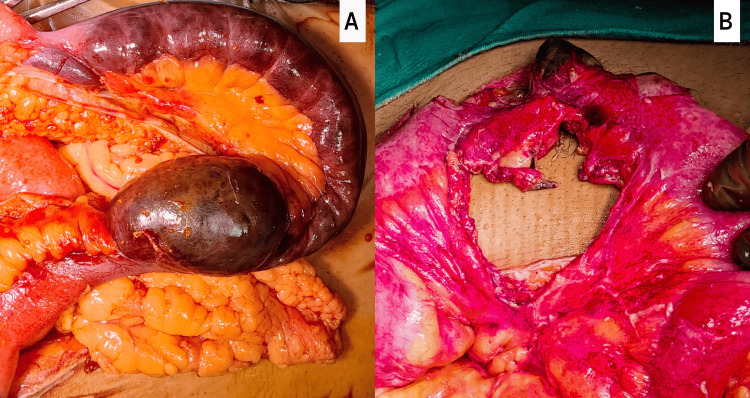
An intraoperative picture showing (A) a gangrenous bowel segment; (B) a resection anastomosis being done.

Intraoperatively, approximately 2.5 feet of the ileal segment were found to be gangrenous. The adjacent small bowel mesentery was found to be dusky with palpable thrombosed mesenteric vessels. The resection of the gangrenous segment with ileo-ileal anastomosis was done, as shown in Figure 2.

The postoperative period was uneventful, and the patient was discharged on the ninth day postoperatively. The postoperative period was uneventful. The patient was allowed oral intake on postoperative day seven, which he tolerated well. He was discharged on day nine, and he did well during his follow-up visits to the hospital.

Case 3

A 64-year-old male truck driver presented with acute lower abdomen pain following a road traffic accident. The patient's pulse was 118 beats per minute, and his blood pressure was 116/64 mmHg. Abdominal rigidity and abdominal distension were noted. He was HIV-positive. There were sluggish bowel sounds. There was marked leucocytosis (28,000 cells per cubic millimeter).

Intraoperatively, a transverse tear was noted in the adjacent small bowel mesentery, compromising the blood supply of the small bowel, and 4.5 feet of the ileum were found gangrenous. Resection of the gangrenous segment and double barrel ileostomy were done.

The postoperative period was uneventful. The patient was allowed oral intake on postoperative day eight, which he tolerated well. He was discharged on day ten, and he did well during his follow-up visits to the hospital.

## Discussion

Although mortality rates of acute mesenteric ischemia (AMI) have been on the decline since the 1960s, the mortality range is still substantial, occurring at rates between 60% and 80% [7]. The exact AMI categorization has an impact on the likelihood of death, with acute thrombosis and acute thromboembolism having the best prognosis. Advanced age, bowel resection during second-look surgery, metabolic acidosis, renal failure, and illness duration are all linked to increased mortality [8]. Since the pathophysiological form of AMI is associated with a number of clinical circumstances, a thorough history is crucial [9]. Chronic postprandial pain in the abdomen, weight loss that is progressing, and past revascularization procedures for mesenteric artery occlusion are all typical among patients with mesenteric arterial thrombosis. Patients with non-occlusive mesenteric ischemia typically have more diffuse, episodic pain that is accompanied by inadequate cardiac activity [10]. Abdominal cramps, nausea, vomiting, and diarrhea constitute typical symptoms of mesenteric vein thrombosis in such patients.

AMI might necessitate the ordering of several laboratory tests; however, these tests are unlikely to confirm the diagnosis. Some investigations focused on laboratory tests including lactate and D-dimer levels, base excess, leukocyte count, and specific biomarkers like alpha-glutathione S-transferase and intestinal fatty acid binding protein (I-FABP) [11]. According to recent studies, radiological examination is more crucial. A plain X-ray of the abdomen may not reveal free gas under the diaphragm even if there is bowel gangrene in the absence of bowel perforation. Ultrasonography may show dilated bowel loops with echogenic peritoneal collection. Multi-detector CT angiography has established itself as the gold standard for the diagnosis of AMI due to its high sensitivity (94%) and specificity (95%) [12]. The method of treatment varies depending on the underlying cause of mesenteric ischemia; nonocclusive AMI is managed medically, but occlusive AMI requires surgery. Regardless of the cause, any patient with mesenteric ischemia and symptoms of peritonitis or potential bowel infarction needs to have immediate surgery to remove any ischemic or necrotic bowel.

The present series reports three patients with intestinal gangrene following a road traffic accident. The underlying cause of intestinal ischemia in those patients was a transverse tear in the mesentery of the small intestine, leading to the disruption of branches of the SMA. The adjacent small intestine soon became gangrenous. The diagnosis is that all patients were bonded on the triad of severe abdominal pain following a road traffic accident, the absence of pneumoperitoneum, and the presence of echogenic collection suggestive of hemoperitoneum on ultrasonography. It is clear that AMI caused by SMA disruption is a dangerous and potentially fatal syndrome, and once intestinal necrosis occurs, the prognosis for this condition will be poor. In order to effectively assess patients, focused early diagnosis, prompt action, and supportive intensive care need to be provided.

## Conclusions

Considering the rarity of mesenteric thrombosis following trauma, this case series emphasizes the significance of taking arterial thrombosis into account when making a differential diagnosis for a patient who has experienced blunt trauma. In contrast to arterial thromboembolism, venous thromboembolism appears more frequently in the literature as a known consequence of trauma. In order to achieve optimum outcomes in such patients, there should be adequate investigation, early intervention, and rapid control of AMI. There should also be a high level of suspicion for the proper management of such patients.
